# Supplementary material for: PRMT5 is essential for B cell development and germinal center dynamics
Source: Nat Commun. 2019 Jan 3;10:22. doi: 10.1038/s41467-018-07884-6 (PMC6318318; doi:10.1038/s41467-018-07884-6)
Supplement: Supplementary file 1 — Supplementary Information [file 41467_2018_7884_MOESM1_ESM.pdf]

Supplementary Information

PRMT5 is essential for B cell development and germinal center dynamics

Litzler et al.

Supplementary Figures

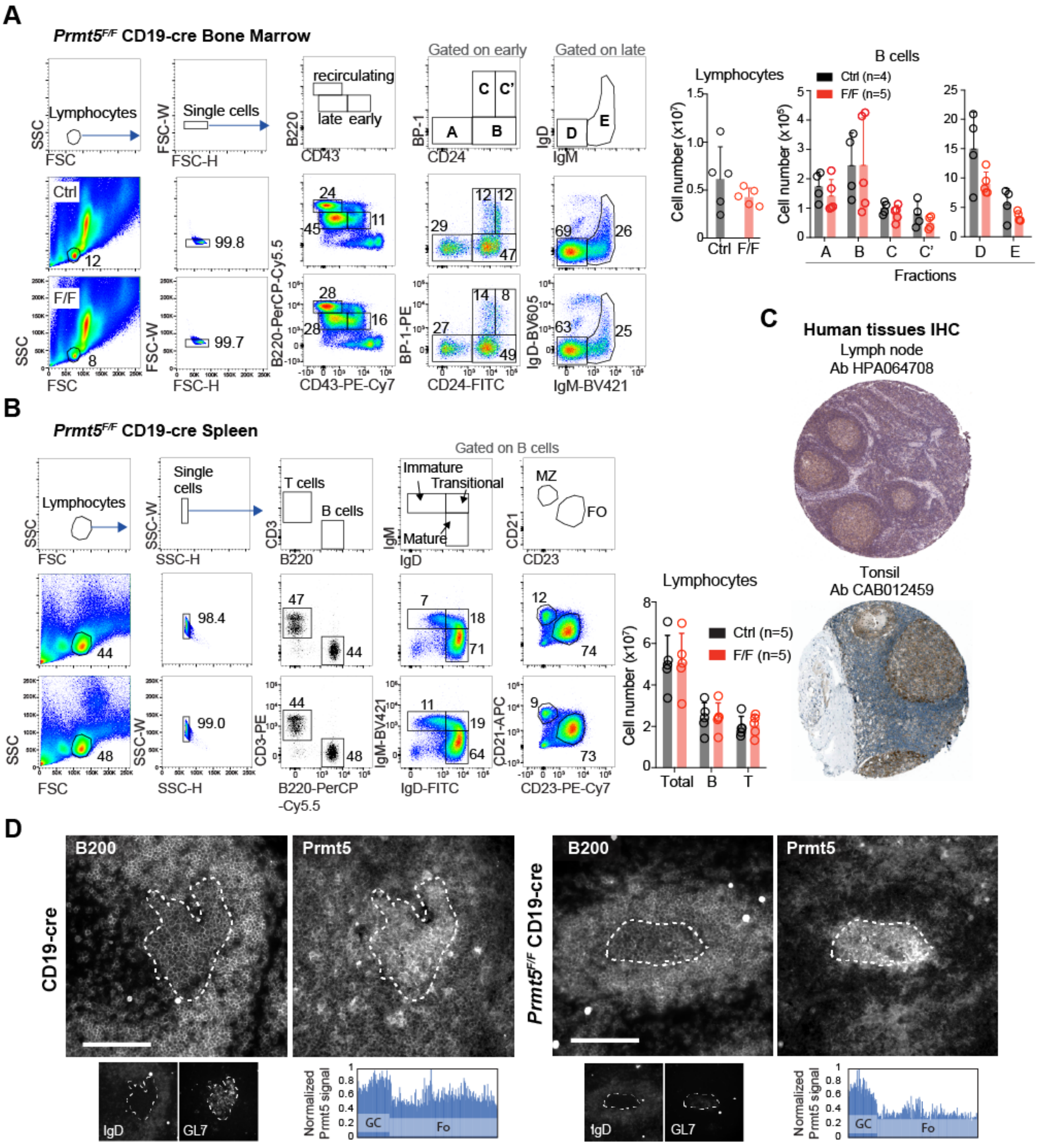

Supplementary Figure 1 – Normal B cell development in *Prmt5*$^{F/F}$ CD19-cre mice.

A) Gating strategy and representative flow cytometry for BM lymphocytes and Hardy's fractions of B cell development in CD19-cre (Ctrl) and *Prmt5*$^{F/F}$ CD19-cre (F/F) mice. Plots show means + s.d. of absolute lymphocyte and B cell fractions counts for 5 mice (3-4 months of age) per genotype from one experiment. The same gating strategy was used in Fig. 7. **B)** Gating strategy

and representative flow cytometry splenic lymphocytes and B cell subpopulations quantified in Fig. 1C: immature ($IgD^- IgM^+$), transitional ($IgD^+ IgM^+$), mature ($IgD^+ IgM^{low}$), follicular ($CD21^+ CD23^+$) and marginal zone ($CD21^+ CD23^-$). Plots show means + s.d. of absolute splenic lymphocytes and B cell subsets counts in the mice from A). **C)** Immunohistochemistry pictures of human lymph node (LN) and tonsils stained for Prmt5 with the indicated antibodies as extracted from the Human protein atlas database (www.proteinatlas.org). **D)** Representative immunofluorescence pictures of B cell follicles containing GC in splenic sections from the indicated mice at day 14 post-NP-CGG immunization, stained for the indicated antigens. GC were traced based on simultaneous IgD and GL7 staining. Scale bar, 100 μm. The histograms show the distribution of normalized Prmt5 signal for all individual cells in the GC ($B220^+ IgD^- GL7^+$) and the mantle zone (Fo) ($B220^+ IgD^+ GL7^-$).

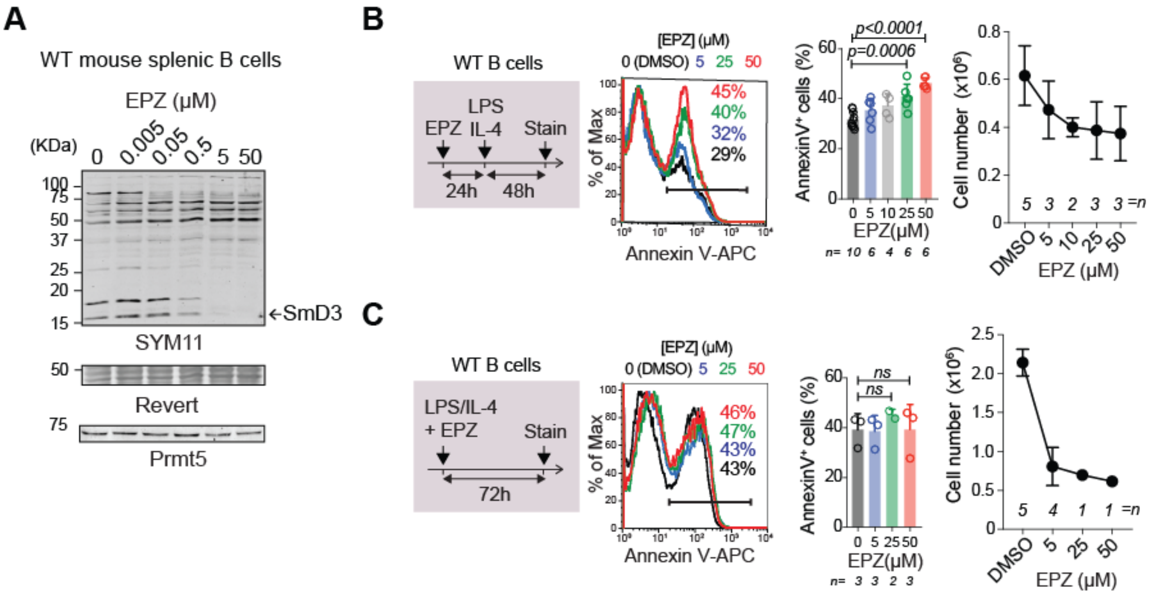

Supplementary Figure 2 – B cell apoptosis depends on the timing of Prmt5 inhibition

A) WB for sDMA (SYM11) and Prmt5 in extracts from WT splenic B cells stimulated with LPS (5 µg/mL) and IL-4 (5 ng/mL) for 72 h in the presence of the indicated concentration of Prmt5 inhibitor EPZ015666 (EPZ). **B)** Experimental set up and representative flow cytometry histogram of the proportion of Annexin-V$^+$ B cells that were plated and treated with the indicated EPZ doses for 24 h before activation with LPS (5 µg/mL) and IL-4 (5 ng/mL). Means + s.d. proportion of Annexin-V$^+$ B cells from n mice from 5 experiments are plotted. The rightmost plot shows mean + s.d. cell counts (except for the 10 µM dose, mean ± s.e.m) of n mice from 5 experiments at 72 h post-plating. Significant P-values by one-way ANOVA with Dunnett's correction for multiple comparisons are indicated. **C)** Experimental set up for simultaneous Prmt5 inhibition and B cell activation. Apoptosis and cell count from n mice from 2 experiments were measured and plotted as in B).

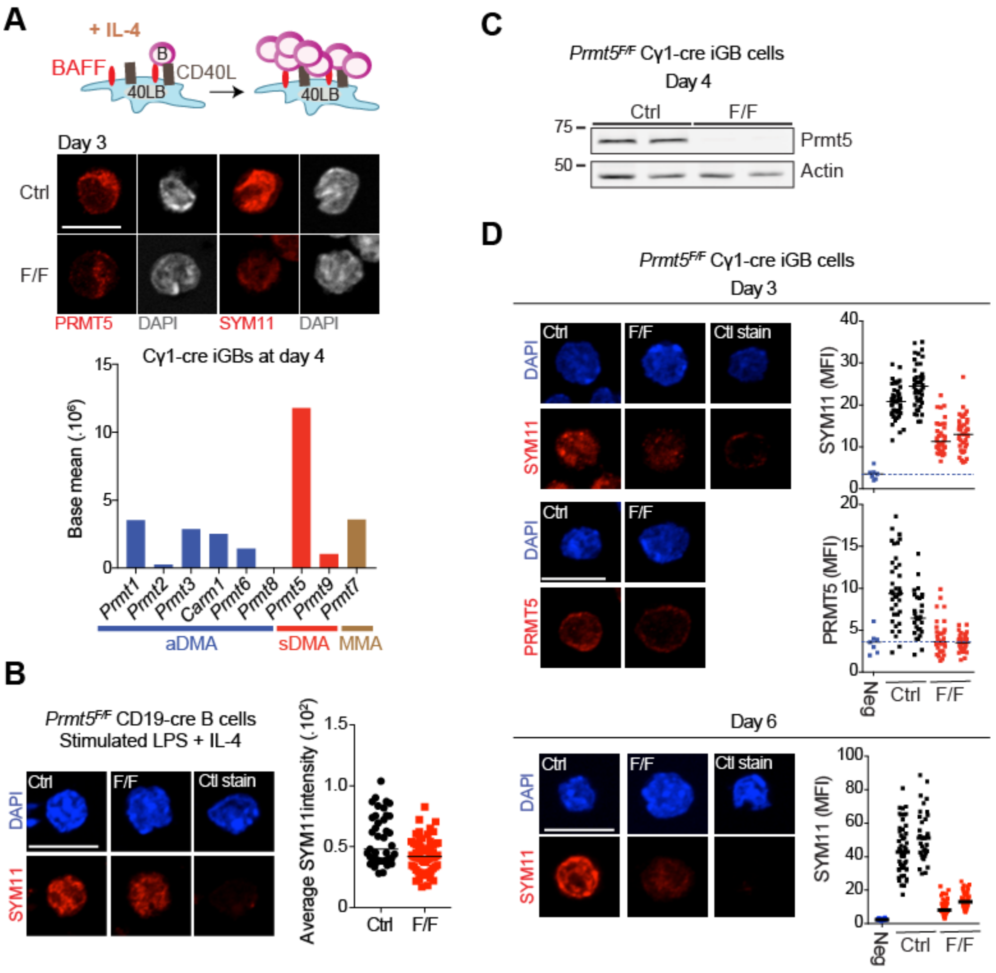

Supplementary Figure 3 –Prmt5 depletion in *Prmt5$^{F/F}$* CD19-cre and *Prmt5$^{F/F}$* Cγ1-cre iGBs

A) Cartoon of the induced GC-like B cells (iGBs) system. Resting splenic B cells were plated on 40LB cells (expressing CD40L and BAFF) and the media supplemented with 1 ng/mL IL-4. Representative IF of sDMA (SYM11), Prmt5 and DAPI on Cγ1-cre (Ctrl) and *Prmt5$^{F/F}$* Cγ1-cre (F/F) iGBs at days 3 post-plating are shown. Transcript levels of each Prmt by RNA-seq in control Cγ1-cre iGBs at day 4 post-plating are shown (average of 4 biological replicates). **B)** Representative IF of sDMA (SYM11) and DAPI on CD19-cre (Ctrl) and *Prmt5$^{F/F}$* CD19-cre (F/F) iGBs at day 6 post-plating. The average SYM11 intensity per cell (symbols) and mean intensity per genotype (bars) are plotted for a pool of 2 mice per genotype. **C)** Representative WB of Prmt5 and Actin, as a loading control, in extracts of iGB cells from Cγ1-cre (Ctrl), *Prmt5$^{F/F}$* Cγ1-cre mice (F/F) at day 4 post-plating. **D)** Representative IF of sDMA (SYM11), Prmt5 and DAPI on Cγ1-cre (Ctrl) and *Prmt5$^{F/F}$* Cγ1-cre (F/F) iGBs at days 3 and 6 post-plating. The average SYM11 and Prmt5 intensity per cell (symbols) and mean per genotype (bars) are plotted for 2 mice per genotype. Scale bars = 10 μm in C and E.

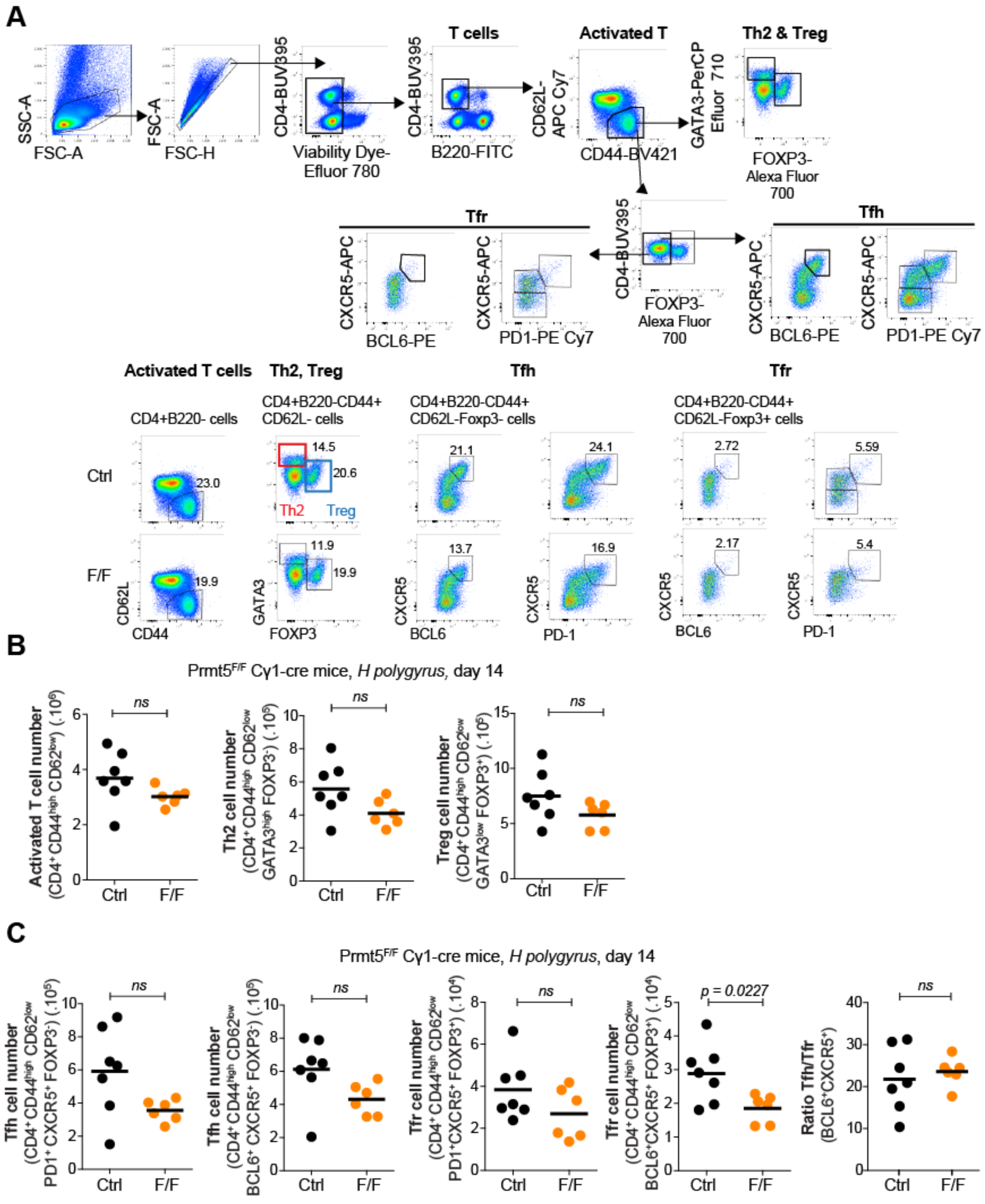

Supplementary Figure 4 – T cell populations in mice infected with *H. polygyrus*

A) Gating strategy for analyzing T cell subsets and representative results. **B)** Compiled absolute numbers of T cell populations in Cγ1-cre (Ctrl) or *Prmt5$^{F/F}$* Cγ1-cre (F/F) individual mice (symbols) and mean values (bars) .14 days after infection with *H polygyrus* from 2 experiments. **C)** Tfh and Tfr populations in the mice from B) were measured using two different methods, differing in the use of PD1 or BCL6 staining were used. The Tfh to Tfr ratio was calculated from the second method. Data from 2 experiments, individual mice (symbols) and mean values (bars) are plotted. P-values by unpaired, two tailed Student-t test.

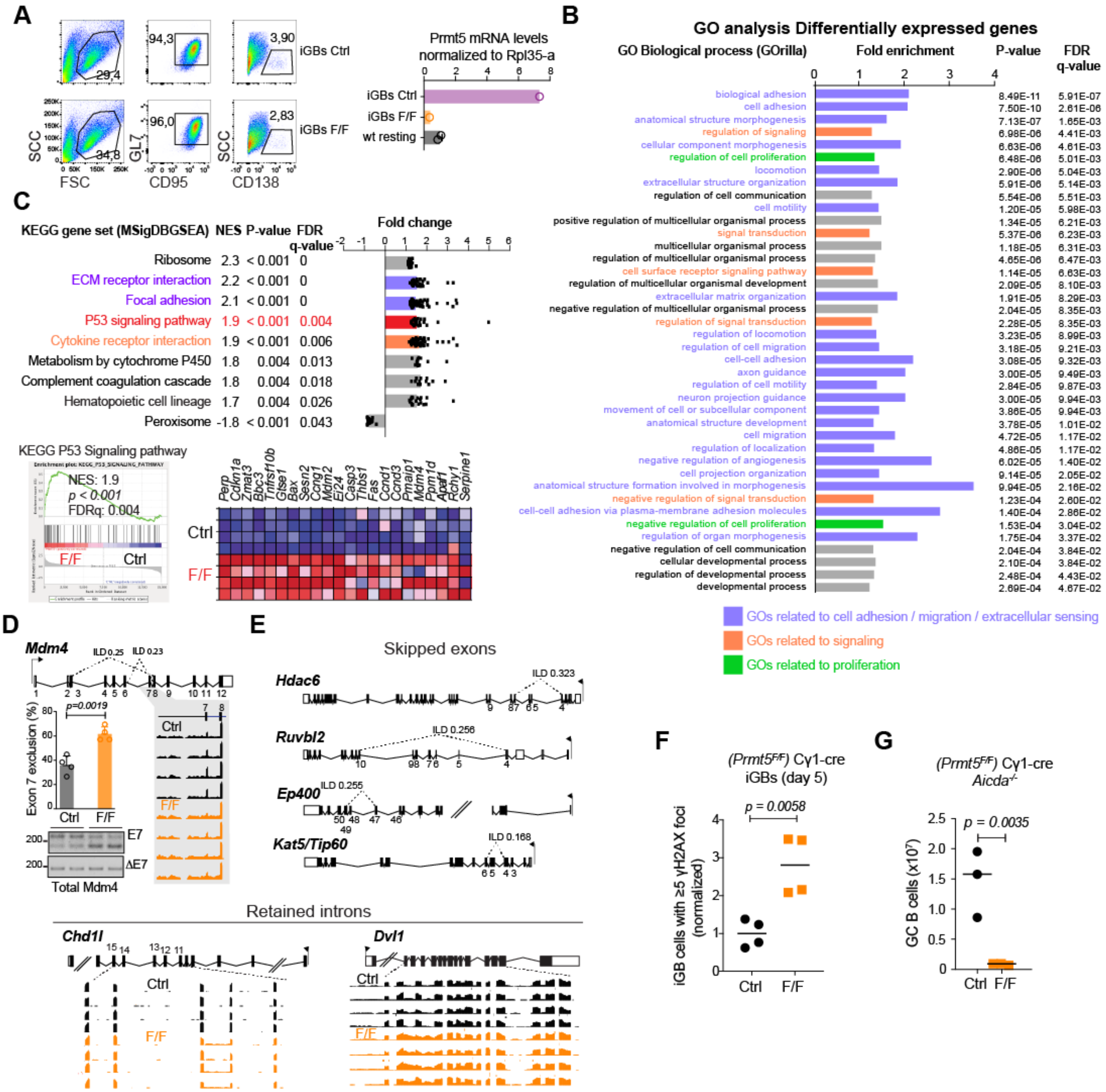

Supplementary Figure 5 – RNA-seq and p53 response additional analyses

A) Representative flow cytometry plots for GC and plasma cell markers in iGB cells derived from Cγ1-cre (Ctrl) or *Prmt5*$^{F/F}$ Cγ1-cre (F/F) splenic B cells at day 4 after plating onto 40LB, as were used for RNA-seq. Prmt5 transcript levels in the same cells compared to resting wt B cells determined by RT-qPCR are plotted. **B)** Functional annotation of differentially expressed genes by GO terms describing biological processes. A list of all genes ranked by fold-change (from maximum to ≥0) was analyzed at the GOrilla server. GO terms with P<0.05 and FDRq value <0.05 were considered significant. **C)** Pathways significantly enriched or depleted in Prmt5-null iGBs by Gene set enrichment analysis (GSEA) against the MsigDB of KEGG pathways. Normalized enrichment score (NES) of pathways with P-value<0.05 and FDRq value <0.05 are shown. The fold-change of genes in the leading edge (symbols) and the median fold change (bars) of each gene set are plotted. The GSEA plot for and heat map of gene expression in the leading edge for KEGG P53 signaling pathway are shown below for all samples. ECM,

extracellular matrix. **D)** Mdm4 gene scheme and alignment of RNA-seq data from each sample with the aligned reads for one SE event in each sample. Representative RT-PCR validating the same event and mean + s.d. quantification of exon 7 exclusion for 4 mice are shown. **E)** Scheme of selected genes with SE events indicating the inclusion level difference (ILD) for significant events and with RI events, including the RNA-seq data alignment of the relevant region. **F)** Mean proportion of iGB cells with ≥5 γH2AX foci determined by IF 5 days after plating. Values for individual Cγ1-cre (Ctrl) and *Prmt5*$^{F/F}$ Cγ1-cre (F/F) mice from 2 experiments are plotted, normalized to the mean of the controls in each experiment. P-value by unpaired, two tailed Student-t test. **G)** Number of GC B cells in the spleen of *Aicda*$^{-/-}$ Cγ1-cre (Ctrl) and *Aicda*$^{-/-}$ *Prmt5*$^{F/F}$ Cγ1-cre (F/F) mice (symbols), 10 days after immunization with SRBC. Data from one experiment.

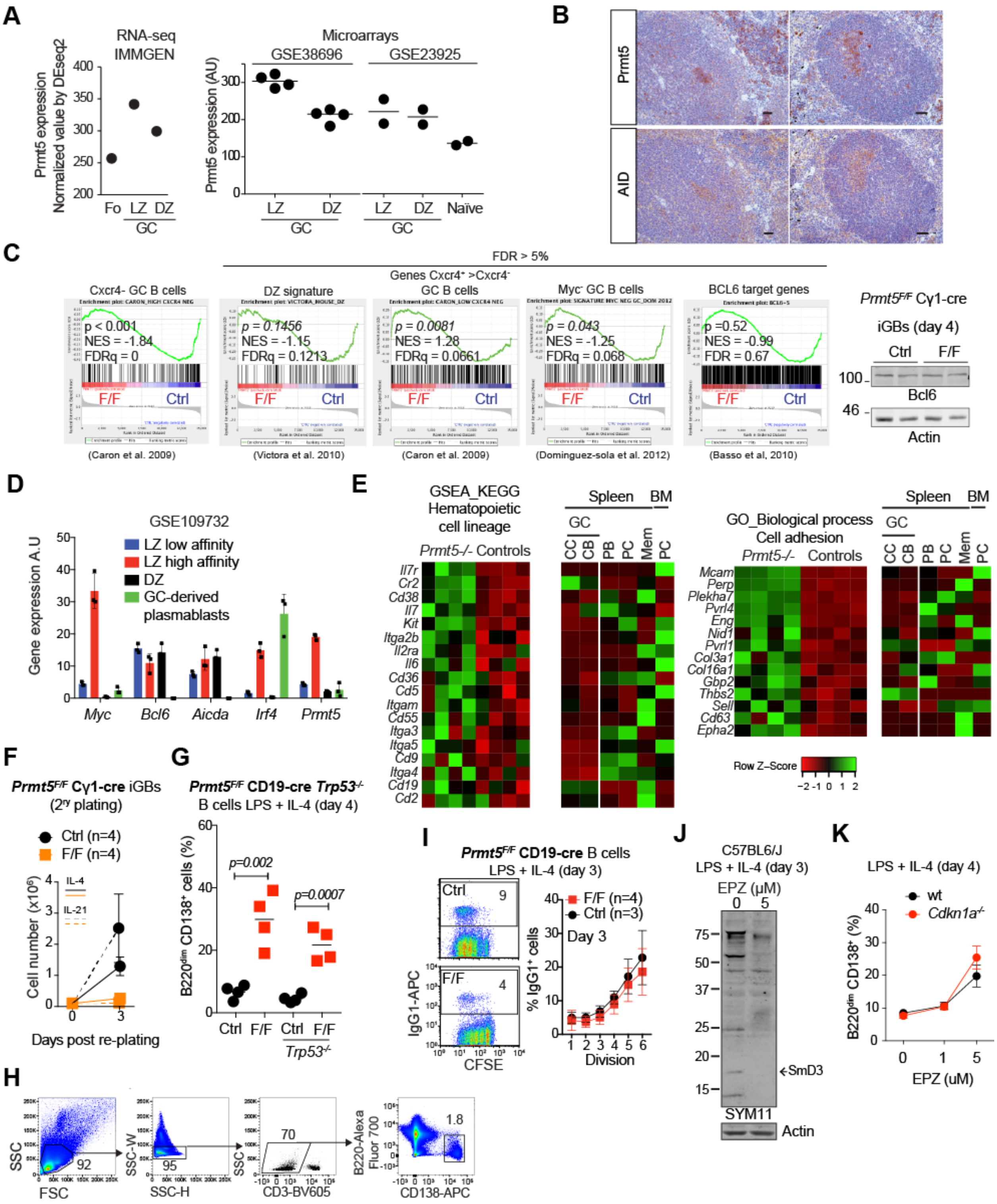

Supplementary Figure 6 – Expression of Prmt5 in GC light zone B cells

A) Relative Prmt5 transcript levels in GC light zone (LZ) and dark zone (DZ) according to gene expression data obtained from the indicated sources. Follicular (Fo) and naïve B cells data are

shown for comparison. **B)** Additional IHC of consecutive splenic sections stained with anti-AID and anti-Prmt5, from immunized C57BL6/J mice. Scale bars = 20 μm (left) and 50 μm (right). **C)** GSEA of transcriptional changes in *Prmt5*$^{F/F}$ Cγ1-cre (F/F) versus control iGB cells using gene sets obtained from the indicated references. Enrichment was considered significant when P<0.05 and FDRq value <0.05. WB probed for Bcl6, and Actin as a loading control, of *Prmt5*$^{F/F}$ Cγ1-cre (F/F) and Cγ1-cre control iGB extracts harvested at day 4. **D)** Relative genes transcript levels between GC subsets in RNA-seq data from the indicated source. Means + s.d. of triplicates. **E)** Gene expression heat maps for each RNA-seq replicate from *Prmt5*$^{F/F}$ Cγ1-cre (Prmt5$^{-/-}$) and Cγ1-cre (Control) iGBs. *Left*, genes from the leading-edge of the "Hematopoietic cell lineage" KEGG pathway gene set. *Right*, Genes most expressed (basemean >100) and more differentially expressed genes in the "Cell adhesion" GO term. The relative expression the same genes in splenic centrocytes (CC), centroblasts (CB), plasmablasts (PB), plasma cells (PC), memory B cells (Mem) and BM plasma cells was obtained from Immgen RNA-seq data and shown as heat map next to each gene set. **F)** Mean ± s.d. iGB cell count at re-plating from 2 experiments comparing Cγ1-cre (Ctrl) or *Prmt5*$^{F/F}$ Cγ1-cre (F/F) iGB cultures. **G)** Plasma cell proportion in cultures of splenic B cells from individual mice (symbols) of the indicated genotypes and mean (bars) for 2 experiments are plotted. **H)** Flow cytometry gating strategy for splenic plasma cells (related to Fig. 9F). **I)** Representative flow cytometry plots of Cγ1-cre (Ctrl) or *Prmt5*$^{F/F}$ CD19-cre (F/F) B cells activated *ex vivo* stained with CFSE and anti-IgG1. Means ± s.d. proportion of IgG1^{+} cells per division, determined from CFSE peaks, are plotted for n mice from 2 experiments. **J)** WB for sDMA (SYM11) and Actin, as a loading control, in extracts from wt splenic B cells stimulated with LPS and IL-4 for 72 h in the presence of 5 μM EPZ. The position of splicing factor Smd3 is indicated. **K)** Mean + s.d. plasma cell proportion in cultures of splenic B cells from wt or p21-deficient mice treated with DMSO or doses of Prmt5 inhibitor EPZ.

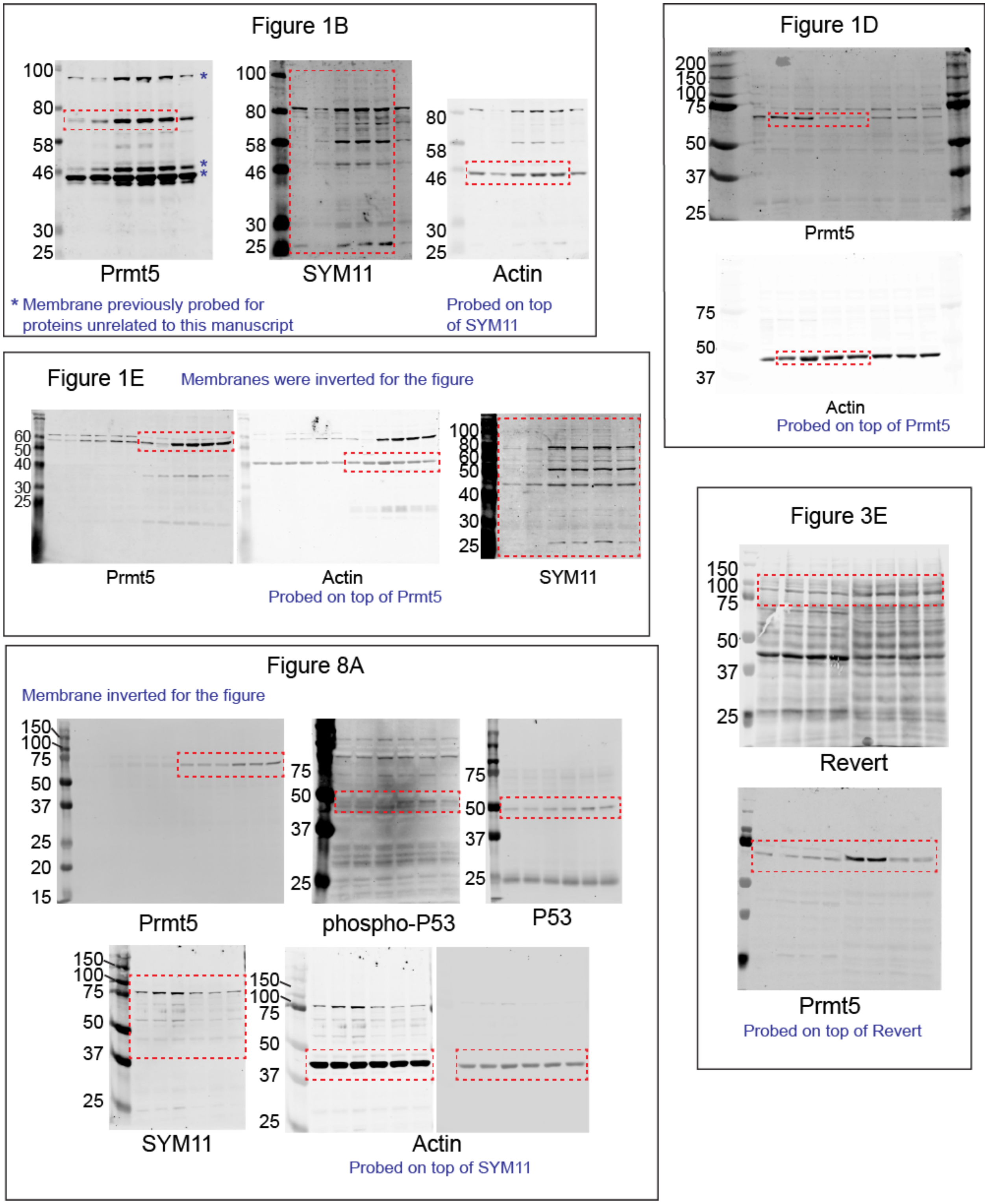

Supplementary Figure 7 – Uncropped Western blots.

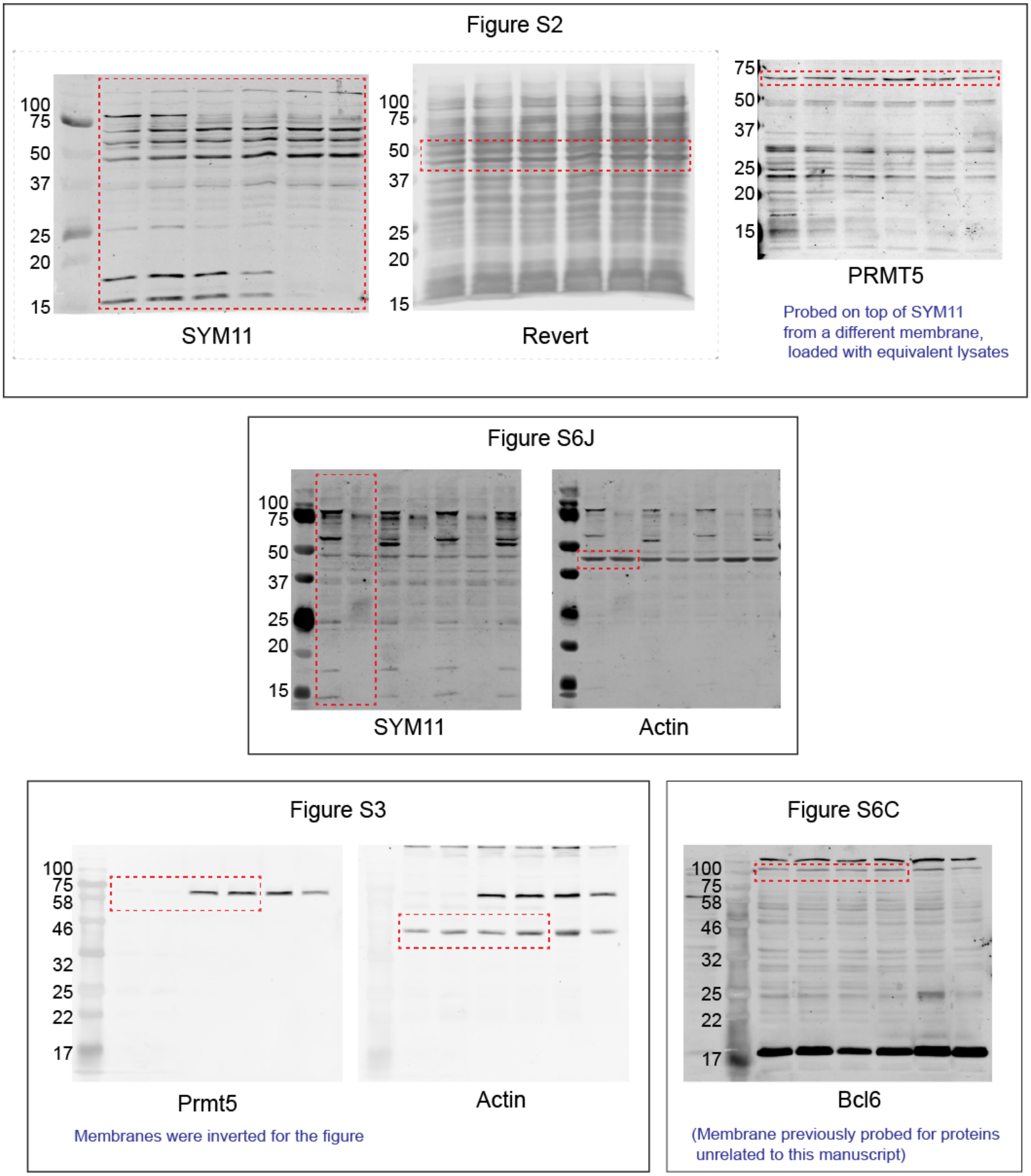

Supplementary Figure 7 – Uncropped Western blots (cont.).

The whole western blots used to construct the indicated figure panels are sohwn, indicating molecular mass markers position and the region cropped from each. Where indicated, the blots were successively probed with different antibodies, so multiple signals are visible; some of which were not used for this manuscript.
