# Supplementary material for: PRMT5 is essential for B cell development and germinal center dynamics
Source: Nat Commun. 2019 Jan 3;10:22. doi: 10.1038/s41467-018-07884-6 (PMC6318318; doi:10.1038/s41467-018-07884-6)
Supplement: Supplementary file 3 — Description of Additional Supplementary Files [file 41467_2018_7884_MOESM3_ESM.pdf]

Description of Additional Supplementary Files

Supplementary Data 1 :

Gene expression changes in Cg1-cre Prmt5F/F iGBs versus Cg1-cre controls.

Supplementary Data 2 :

Functional annotation of gene expression changes in Cg1-cre Prmt5F/F iGBs.

Supplementary Data 3 :

Genes with splicing alterations in Cg1-cre Prmt5F/F vs Cg1-cre iGBs.

Supplementary Data 4 :

Functional annotations of genes with splicing defects in Cg1-cre Prmt5F/F iGBs

Supplementary Data 5 :

Antibody list and details.

Supplementary Data 6 :

PCR primers list.
