# Supplementary material for: PRMT5 is essential for B cell development and germinal center dynamics
Source: Nat Commun. 2019 Jan 3;10:22. doi: 10.1038/s41467-018-07884-6 (PMC6318318; doi:10.1038/s41467-018-07884-6)
Supplement: Supplementary file 10 — Reporting Summary [file 41467_2018_7884_MOESM10_ESM.pdf]

nature research

Corresponding author(s): Javier Di Noia

Reporting Summary

Nature Research wishes to improve the reproducibility of the work that we publish. This form provides structure for consistency and transparency in reporting. For further information on Nature Research policies, see Authors & Referees and the Editorial Policy Checklist.

Statistical parameters

When statistical analyses are reported, confirm that the following items are present in the relevant location (e.g. figure legend, table legend, main text, or Methods section).

n/a	Confirmed	
☐	☒	The exact sample size (n) for each experimental group/condition, given as a discrete number and unit of measurement
☐	☒	An indication of whether measurements were taken from distinct samples or whether the same sample was measured repeatedly
☐	☒	The statistical test(s) used AND whether they are one- or two-sided<br>*Only common tests should be described solely by name; describe more complex techniques in the Methods section.*
☒	☐	A description of all covariates tested
☐	☒	A description of any assumptions or corrections, such as tests of normality and adjustment for multiple comparisons
☐	☒	A full description of the statistics including central tendency (e.g. means) or other basic estimates (e.g. regression coefficient) AND variation (e.g. standard deviation) or associated estimates of uncertainty (e.g. confidence intervals)
☐	☒	For null hypothesis testing, the test statistic (e.g. F, t, r) with confidence intervals, effect sizes, degrees of freedom and P value noted<br>*Give P values as exact values whenever suitable.*
☒	☐	For Bayesian analysis, information on the choice of priors and Markov chain Monte Carlo settings
☒	☐	For hierarchical and complex designs, identification of the appropriate level for tests and full reporting of outcomes
☒	☐	Estimates of effect sizes (e.g. Cohen's d, Pearson's r), indicating how they were calculated
☐	☒	Clearly defined error bars<br>*State explicitly what error bars represent (e.g. SD, SE, CI)*

Our web collection on statistics for biologists may be useful.

Software and code

Policy information about availability of computer code

Data collection	Details provided in the methods section
Data analysis	Details provided in the methods section

For manuscripts utilizing custom algorithms or software that are central to the research but not yet described in published literature, software must be made available to editors/reviewers upon request. We strongly encourage code deposition in a community repository (e.g. GitHub). See the Nature Research guidelines for submitting code & software for further information.

Data

Policy information about availability of data

All manuscripts must include a data availability statement. This statement should provide the following information, where applicable:

- Accession codes, unique identifiers, or web links for publicly available datasets
- A list of figures that have associated raw data
- A description of any restrictions on data availability

Data availability: RNA-seq data generated in this work was deposited at NCBI under accession number GSE120309. Accesion number of RNA-seq or microarray datasets obtained from public repositories are provided in the corresponding figure or figure legend. All other relevant data is included.

Field-specific reporting

Please select the best fit for your research. If you are not sure, read the appropriate sections before making your selection.

☒ Life sciences ☐ Behavioural & social sciences ☐ Ecological, evolutionary & environmental sciences

For a reference copy of the document with all sections, see nature.com/authors/policies/ReportingSummary-flat.pdf

Life sciences study design

All studies must disclose on these points even when the disclosure is negative.

Sample size	Sample size was based on the combination of the magnitude of effect observed, whether or not statistical significance was reached, as well as availability of mice. For most experiments cells from at least 4 mice per group from 2 independent experiments were analyzed.
Data exclusions	No data was excluded from the analysis.
Replication	Experiments were repeated at least twice with at least two animals per experiment per genotype. When both experiments were consistent this was considered sufficient for a conclusion. In very few cases one experiment was conducted, but, as explained in the answer to reviewers, in this case multiple independent evidences supported the conclusions.
Randomization	Mice 2-4 month old were used, mix of males and females that were randomly assigned to experiments according only to their genotype.
Blinding	Blinding was not performed. It was not considered necessary because most measurements were done using objective methods (i.e. via automated software).

Reporting for specific materials, systems and methods

Materials & experimental systems

n/a	Involved in the study
☒	☐ Unique biological materials
☐	☒ Antibodies
☐	☒ Eukaryotic cell lines
☒	☐ Palaeontology
☐	☒ Animals and other organisms
☒	☐ Human research participants

Methods

n/a	Involved in the study
☒	☐ ChIP-seq
☐	☒ Flow cytometry
☒	☐ MRI-based neuroimaging

Antibodies

Antibodies used	Described in Supplementary Data 5
Validation	All antibodies were from commercial sources. Validation was assumed from supplier statement but for most antibodies it was confirmed based on known staining profile and/or the presence of appropriate negative controls (i.e. Cell types or extracts lacking the target antigen)

Eukaryotic cell lines

Policy information about cell lines

Cell line source(s)	40LB cells were generated and provided by Dr D Kitamura
Authentication	40LB cells were authenticated based on the presence of antibiotic resistances and function (capacity to generate iGBs)
Mycoplasma contamination	40LB cells were regularly tested for and confirmed to be Mycoplasma free.
Commonly misidentified lines (See ICLAC register)	none used

Animals and other organisms

Policy information about studies involving animals; ARRIVE guidelines recommended for reporting animal research

Laboratory animals	Mouse lines were all in the C57BL6/J background.
Wild animals	None
Field-collected samples	None

Flow Cytometry

Plots

Confirm that:

☒ The axis labels state the marker and fluorochrome used (e.g. CD4-FITC).

☒ The axis scales are clearly visible. Include numbers along axes only for bottom left plot of group (a 'group' is an analysis of identical markers).

☒ All plots are contour plots with outliers or pseudocolor plots.

☒ A numerical value for number of cells or percentage (with statistics) is provided.

Methodology

Sample preparation	Data provided in the methods section
Instrument	Data provided in the methods section
Software	Data provided in the methods section
Cell population abundance	Data provided in the methods section
Gating strategy	Data provided in main and supplementary figures.

☒ Tick this box to confirm that a figure exemplifying the gating strategy is provided in the Supplementary Information.
